# Development, implementation and evaluation of the ‘BELIEVE’ program for improving physical activity among women: a mixed method action research study

**DOI:** 10.1186/s13102-021-00367-0

**Published:** 2021-10-26

**Authors:** Leila Amiri-Farahani, Soroor Parvizy, Eesa Mohammadi, Mohsen Asadi-Lari, Ziba Taghizadeh, Sally Pezaro

**Affiliations:** 1 Department of Midwifery, Faculty of Nursing and Midwifery, Iran University of Medical Sciences, Tehran, Iran; 2 Department of Pediatric Nursing, Faculty of Nursing and Midwifery, Iran University of Medical Sciences, Tehran, Iran; 3 Department of Nursing, Faculty of Medical Sciences, Tarbiat Modares University, Tehran, Iran; 4 Oncopathology Research Centre, Department of Epidemiology, School of Public Health, Iran University of Medical Sciences, Tehran, Iran; 5 Faculty Member of Nursing and Midwifery Care Research Center, Nursing and Midwifery School, Tehran University of Medical Sciences, Tehran, Iran; 6 Faculty of Health and Life Sciences, Coventry University, Coventry, UK

**Keywords:** Physical activity, Action research, Women, MAPP process, Peer mentors, Advertising available sport services, Cognitive behavioral therapy

## Abstract

**Background:**

There is insufficient physical activity among women. Yet the implementation of effective, multilevel, and evidence-based interventions may address this. Since the lifestyle of individuals is formed in many different social, physical and cultural contexts, it will be necessary in designing such interventions to involve many stakeholders. Consequently, the present study took a mixed method action research approach in developing, implementing and evaluating a bespoke program to improve physical activity among women.

**Methods:**

This study was conducted within the Khoramroudi neighborhood of Tehran between 2013 and 2015 utilizing the four main phases of action research. The Mobilizing for Action through Planning and Partnerships (MAPP) process was used to design the study. During the initial phase, participants were organized into three groups; a core support committee, a steering committee, and a study population. Qualitative and quantitative data were also collected during this first phase. During the second phase, interventions were developed and implemented. In the third phase, an evaluation was carried out using both quantitative (Designing a quasi-experimental study) and qualitative methods. During the fourth phase, an exploration of the structure and process of action research was completed with the aim of providing a conceptual model and descriptions of the context.

**Results:**

Three strategic interventions were effective in improving physical activity among women: (1) utilization of sports assistants; (2) Local health promotion and the dissemination of an informational, motivational and culturally competent booklet entitled “Educational content for sport assistants" (3) Group-based cognitive behavioral therapy. Quantitative results [Significant difference between the total score of PA before the intervention, and 1 and 3 months after the intervention (*P* < 0.001)] and the results of qualitative evaluations were shown to improve physical activity among participants. The newly co-created “adjusted MAPP model” was offered within three action cycles. The structure of this was described to capture the impacts of interactions among a variety of stakeholders.

**Conclusion:**

The comprehensive identification of problems led to the development of collaborative strategies. Strategies of action research can positively affect physical activity among women. To improve physical activity outcomes more generally, the use of MAPP principles and strategies is suggested to meet the specific needs and strengths of all community members.

## Background

Insufficient physical activity (PA) is a high-risk behavior and a risk factor for morbidity and mortality in the world [1]. For adults aged 18–65, participation in aerobic physical activities for at least 30 min, 5 days a week is recommended [2]. Yet based on the results of Urban HEART (Health Equity Assessment and Response Tool) project, the level of PA among men and women living in all areas of Tehran, in Iran, is 20.5% and 24.3%, respectively [3]. This study also revealed that obstacles to PA for women in particular included childcare responsibilities, out-of-home work, security concerns, inability to cover financial costs, dressing and makeup, and religious beliefs [4–6]. For example, due to wearing a hijab outdoors, many Muslim women in the warmer climates of Iran may prefer to exercise indoors, access to which is not always available [7]. Consequently, there is a need to implement and evaluate bespoke and evidence-based PA interventions to support this unique population.

As Iran is predominantly an Islamic country, many people have a unique cultural point of view from those in other countries [8]. For example, in practising the Islamic faith, some women are expected to wear modest clothing; including clothing that covers most of the body [7]. This may in turn impact upon a woman’s ability to engage in PA. Some Muslim women may also choose to wear a hijab to hide their bodies during PA, and disengage from activities which demand body contact with males [9]. Furthermore, the unique weather conditions in Tehran, Iran discourages some from walking and engaging in PA outdoors [10]. A built up environment also lends itself to driving rather than walking, further contributing to reduced PA [11]. As such, adopting an appropriate approach to the promotion of PA which considers the context, barriers and facilitators of PA from the point of view of women in this society is required.

Face-to-face interventions are very common in the promotion of PA [12, 13]. Yet since lifestyles are shaped within a variety of different social, physical, and cultural contexts, multi-level approaches which consider cultural, environmental, and personal factors may be more successful than individual programs [14]. Such approaches are effective as they can promote health and make changes at one or more levels with an ecological focus [15]. Interventions also provide cost-effective strategies to promote health through which a large number of people are able to use [16–18]. Consequently, alongside women, it will also be important in the implementation and evaluation of any new interventions to consider partnership working with and significant involvement from communities, authorities, and other key stakeholders in the locality. One approach to achieving this is the undertaking of Action Research (AR).

Other international studies on PA interventions have already been guided by an AR approach. For example, Berger and Peerson's study investigated the relationship between PA and the social milieu of young Muslim women in the United Arab Emirates [5]. Set within a context of rapid social change, perceived barriers to daily exercise influenced participants’ PA levels and overall well-being. Results indicated a lack of physical exercise and strategies were proposed for implementation by college staff and students [5]. Jacob’s AR with the aim of increasing physical fitness and fitness attitudes through choice and student designed fitness activities showed no notable change in the students’ attitude toward physical fitness [19]. This could be due to the fact that students believed in the importance in physical fitness from the beginning. What was notable to the teacher researcher was the increase in participation during the day activities, as well as an increase in the variety of fitness activities which the students became interested in [19]. Similarly, Holt et al. [20] undertook AR with the purpose of developing, implementing and evaluating sport-based after-school programs for students in low-income areas of Edmonton, Alberta, Canada. Children reported that they enjoyed activities based on creating optimal challenges and ‘adventures’ which engaged their imaginations. Children also learned fundamental movement, sport and life skills, some of which transferred to other areas of their lives [20]. Here, scores for the domains relating to physical health, psychological health, social relationships and health satisfaction where significantly higher after participation in the PA intervention program compared to the pre-test scores (*p* < 0.05) [21]. These studies and their findings make important contributions to our understandings in relation to the development of intervention programs to promote health equity among citizens in disadvantaged areas. Multiple approaches are required in the evaluation and the feasibility testing of such interventions if they are to be implemented effectively for larger communities in need [5, 19–21].

The aim of the present intervention study was to promote PA in women taking a AR approach. Since the promotion of PA is considered a priority for the women of Tehran, the Khoramroudi health center, affiliated with Tehran municipality was considered the most appropriate intervention study location.

## Methods

To achieve the aim of this action research, it was conducted via four phases: (1) Exploratory; (2) Action; (3) Monitoring and evaluation; (4) Reflection. We recognized that one of the challenges in conducting and reporting such a large RA project such as this is that there is an abundance of activities to articulate. Therefore, an overview of methods for all 4 phases of this action research is presented in Table 1.

**Table 1:** Summary of four phases of action research in women living in Khorramrudi neighborhood based on the MAPP model

*First phase (Exploratory phase)* Identifying the dimensions of the problem and designing strategies for promoting PA			
(1) Determining the members of the working committee	(2) Determining and creating a shared vision	(3) Assessments based on the MAPP model	(4) Identifying strategic priorities for promoting PA
Stakeholders divided into three groups, including: Core research committee (Including the research team), MAPP committee (Including the head of the Khoramroudi sports club, the health directorate of district 2, the sport directorate of district 2, the sport coach of the club and the head of Khoramroudi health center), Research community (Participants, the administrator of the district, and the secretary general of Khoramroudi)	The purpose of this phase was to give an appropriate name to the program and determine the vision for it The research team members, women from Khoramroudi, and the members of the steering committee participated in a focus group discussion	Five major exploratory investigations were carried out: (1) *Determining health priorities of the community and Khoramroudi neighborhood*: An evaluation of the Urban HEART project identified insufficient PA as a significant health problem in district 2 of the Tehran municipality and Khoramroudi neighborhood. Thus, this was considered the most suitable setting for the program (2) *Determining the prevalence of PA in women living in the Khoramroudi neighborhood by* a cross-sectional study. Sampling was performed on 300 non-pregnant women aged between 18 and 65 years old that administered demographic characteristics and IPAQ to women (3) *Agreeing on the importance of PA in women* To reach an agreement on the importance of PA, a FGD was held among the members of the steering committee, the research team members, and the participating women (4) *Identification of barriers and facilitators of PA in women* To achieve this goal, both qualitative and quantitative methods were used. Quantitative section by a cross-sectional study with administering the EBBS. Qualitative section by two FGD with 11 and 13 participants (consisting of women, research team members, and the steering committee) and 6 individual semi-structured interviews were held (5) *Identifying the positive and negative forces for change*. Positive forces and negative forces were identified through interviewing and interacting with the members of the district council, the health center manager, the sport club managers, sport and health officials, the female members of the local sports center and those living in the district	Evidence based stategic priorities were explored via a systematic review of the literature A FGD was held with female participants from the district, the members of the research team, personnel of the Khoramroudi health center, and the health director of the social security department of district 2 of the Tehran municipality The participants were asked to identify strategies that were most important from their point of view, or those which could be resolved using available resources
*Second Phase (Action)* Development and implementation of practical interventions for promotion of PA and Determining action cycles for improvement of PA			
1. Interventions for placement within the programme were co-created via one FGD and 7 individual interviews with all stakeholders 2. Participants acted as co-creators and were asked to provide practical suggestions for the development of a practical intervention and suggest appropriate infrastructures for the implementation of strategies 3. They were also asked to provide details on the time, conditions, how and at what time each intervention should be implemented, and how its progress should be evaluated and monitored 4. Two iterations of a three-step action cycle were conducted (Planning, implementation, and evaluation). This cycle is similar to other continuous improvement cycles based on the spiral of AR involving planning, acting, observing, and reflecting. In both cycles, quantitative and qualitative evaluations were performed			
*Third phase (Monitoring and Evaluation): Assessing the effectiveness of the program quantitatively and qualitatively*			
1. For the quantitative study, this study encompassed a pre and post-test semi-experimental design with the aim of determining and comparing PA before, 1 month and 3 months after the intervention among 70 women to join the study to focus on at least one or more of the following three strategies; peer support, improving knowledge about the sports facilities available in the district, and participating in the cognitive behavioral therapy classes by filling in IPAQ. This phase was repeated with another 70 participants, making a total of 140 participants overall 2. For the qualitative study, a qualitative study was also carried out with the aim of explaining the opinions, perceptions, feelings, and experiences of participants about the process and outcome of PA promotion. All women participated in a total of three FGD’s, which were held over a period of 3 months. Also, semi-structured individual interviews were held to monitor and report the promotion of PA in women who did not participate in the FGDs. Data was analysed thematically			
*Fourth phase (Reflection): Improving strategies to enhance PA in the AR cycles and explaining the process and structure of AR*			
1. *Process explanation* Data was collected from the exploratory phase from beginning to end via the researcher’s daily field notes, audio files of FGDs and related transcriptions. Findings of the FGD’s with women, the research team members and the officials of Health and Sport Administrations of Social Deputy of District 2 of Tehran municipality were then used to inform decision-making and planning. The recommendations of all stakeholders in line with our evidential findings were used to promote PA throughout. For data analysis, directed content analysis was used 2. *Structural explantation* A series of documents, investigations, and internal and external reports were collected and analyzed collectively by the research team members, and a stakeholder group including the social deputy of district 2 in the Tehran municipality, the social deputy of the municipality of Tehran, the district council of Khoramroudi neighborhood and the board of trustees of the district mosques. All correspondences such as formal and informal letters, phone calls, text messages, and e-mails were recorded, and considered as interactions between groups. They were used to develop practical examples of the AR process. The reflections of the Khoramroudi neighborhood’s health authorities, health center, and neighborhood center, as well as the participating women and the results of program evaluation led to the confirmation of the program’s desirability and feasibility			

### First phase (exploratory)

The purpose of this phase was to identify the dimensions of the problem and designing strategies for promoting PA based on the Mobilizing for Action through Planning and Partnerships (MAPP) process. Primarily, the first 4 stages of the MAPP process were followed. These included (1) Determining the members of the working committee to mobilize action (including the head of the Khoramroudi sports club, the health directorate of district 2, the sport directorate of district 2, the sport coach of the club and the head of Khoramroudi health center, four female participants, the administrator of the district, and the secretary general of Khoramroudi); (2) Determining the vision and creating a shared vision among participants (12 women from Khoramroudi neighborhood invited to study by call was installed on the bulletin board of health center); (3) Explaining the results of evaluations to promote PA in women living in the Khoramroudi neighborhood (Four assessments based on the MAPP model); and (4) Identifying strategic priorities for promoting PA (Systematic review to identify best evidence based practices and FDG).

Five major exploratory investigations were also conducted in this first phase. The first enabled us to determine the prevalence of PA in women living in the Khoramroudi neighborhood as we conducted a cross-sectional survey study to determine the prevalence of PA in women living in the Khoramroudi neighborhood. Sampling was performed on 300 non-pregnant women aged between 18 and 65 years old in concurrence with the studies conducted on PA for the Urban HEART project [3], with *P* = 23%, d = 0.05, α = 0.05 and Z = 1.96. Using the ratio estimation formula and considering the probability of a 10% sample drop out, the required sample size was estimated to be 300.

$$\begin{aligned} & n = \frac{{Z^{2} .P(1 - P)}}{{d^{2} }} \\ & n = \frac{{(1.96)^{2} .0.23(1 - 0.23)}}{{(0.05)^{2} }} = 272 \\ \end{aligned}$$

Participants were selected at random from the 51 housing blocks within the neighborhood and recruited via home visits. The survey tool administered included demographic characteristics and the international PA questionnaire (IPAQ). The validity and reliability of the Persian version of this questionnaire have been assessed by Moghadam et al. [22]. The questionnaires were completed by participants after receiving their consent.

The second evaluation enabled us to agree on the importance of PA in women. An FGD was held among the members of the steering committee, the research team and the participating women. The participating women were invited to attend via bulletin board notices within the health center and text messaging. Two further evaluations enabled us to identify barriers and facilitators of PA in women. A survey within a cross-sectional study was performed by administering the Exercise Benefits/Barriers Scale [23]. The validity and reliability of the Persian version of this tool have been confirmed by Farahani et al. [24]. Thereafter, two FGDs and 6 individual semi-structured interviews were held with local participants aged between 18 and 65 years old. The participating women were again invited to attend via bulletin board notices within the health center and text messaging. The interview guide included questions such as ‘what are the barriers and constraints of PA in women?’ And ‘what factors contribute to the promotion of PA in women?’ The duration of individual interviews varied from 35 to 80 min. The duration of each FGD was 60–90 min. Sessions were audio-recorded after obtaining consent. They were then transcribed verbatim, and the coding process began simultaneously with the data collection. Qualitative content analysis [25] was used for data analysis. MAXQDA software was used for the management of data. The trustworthiness of this component was based on Guba’s criteria [26]. Credibility was established through prolonged engagement with the participants and extended immersion in the data. Member checking and peer reviewing were done to verify the findings. A triangulation of the data was performed using field notes and diaries used for data collection. Transferability was facilitated by a purposive sampling of women who identified as having previously experienced physical inactivity. A detailed description of the findings was provided along with a review of the literature to support the study findings. An audit trail was conducted to ensure the confirmability of findings.

Lastly, positive forces (Existing institutional facilities, facilitators, and outsourcing opportunities) and negative forces (Existing barriers and limitations within the organization and external threats) were identified through interviewing and interacting with the members of the district council, the health center manager, the sport club managers, sport and health officials, the female members of the local sports center and those living in the district.

As all decisions in relation to health should be informed by the best available evidence [27], strategic priorities for promoting PA were then explored via a systematic review of the literature and used to stimulate academic discussions. Following this, a further FGD was held with female participants from the district, the members of the research team, personnel of the Khoramroudi health center, and the health director of the social security department of district 2 of the Tehran municipality. Here, the research team reported the findings from previous phases and presented the obstacles and facilitators of PA as well as positive and negative forces for change. Then, the participants were asked to identify strategies that were most important from their point of view, or those which could be resolved using available resources.

### Second phase (action)

As presented in Table 1, the second phase of this action research enabled us to act. Here, we engaged in the development and implementation of practical interventions for the promotion of PA (Co-creation of interventions) and the determining of action cycles for the quantitative and qualitative monitoring and evaluation, and the improvement of PA interventions within the program (Three-step action cycle (Planning, implementation, and evaluation).

### Third phase (monitoring and evaluation)

The third phase of this action research consisted of monitoring and evaluation. This encompassed a quantitative pre and post-test semi-experimental design with the aim of determining and comparing PA before, 1 month and 3 months after the intervention. A qualitative study was also carried out with the aim of explaining the opinions, perceptions, feelings, and experiences of participants about the process and outcome of PA promotion.

#### Quantitative evaluation (conducted with cohort 1)

A pre and post quasi-experimental study was conducted with the aim of examining the effect of designed interventions on the PA of women living in the Khoramroudi neighborhood. Participants were invited to take part if they met the inclusion criteria of being a woman aged between 18 and 65 years old and engaged with insufficient PA. Participants were invited to join the study to focus on at least one or more of the following three strategies; peer support, improving knowledge about the sports facilities available in the district, and participating in the cognitive behavioral therapy classes. The sample size was calculated considering a 95% Confidence Interval (CI) and 80% power and considering the minimum difference or desirable change of Effect size (ES) = 0.5 in each scale between groups and a 10% dropout rate [28]. To assess changes in PA, the IPAQ tool was administered before the intervention period, at the end of the intervention period and 3 months after the intervention period [22].

$$\begin{aligned} & n = \frac{{2(Z_{1} + Z_{2} )^{2} }}{{d^{2} }} \\ & n = \frac{{2(1.96 + 0.84)^{2} }}{{(0.5)^{2} }} = 63 \\ \end{aligned}$$

#### Qualitative evaluation (conducted with cohort 2)

All women participated in a total of three FGD’s, which were held over a period of 3 months. The purpose of these is outlined in Table 1. Sessions were held with the women participating in the research, the research team, a member of the district council, sport assistants, the head of the health center and the head of Khoramroudi neighborhood’s sports club. The length of each FGD was 75–120 min. The interview guide consisted of questions such as: How do you evaluate your exercise experiences? How can you facilitate PA in women? What barriers did you have to exercise and how could these obstacles be removed? Do you think this program has been successful in improving your PA? What suggestions and criticisms do you have regarding the continuance and improvement of the program? Also, semi-structured individual interviews were held to monitor and report the promotion of PA in women who did not participate in the FGDs. The duration of each interview was from 25 to 60 min. Data was analysed thematically. The rigor of this data was assessed in the same way as presented in the first phase (exploratory).

### Fourth phase (reflection)

The final phase of this action research consisted of reflection. To improve strategies to enhance PA in the AR cycles and to explain the process and structure of AR we engaged the Donebedian model in two parts of process (Giving and receiving services) and structure (Context of providing human and other resources) [29]. Participants were also invited to reflect on the MAPP process. The participants in this reflective phase consisted of the research team, the steering committee, and the participants as suggested by the framework of the MAPP process [30]. Data collection was performed in the two stages as follows:

#### Process explanation

**Fig. 1:**
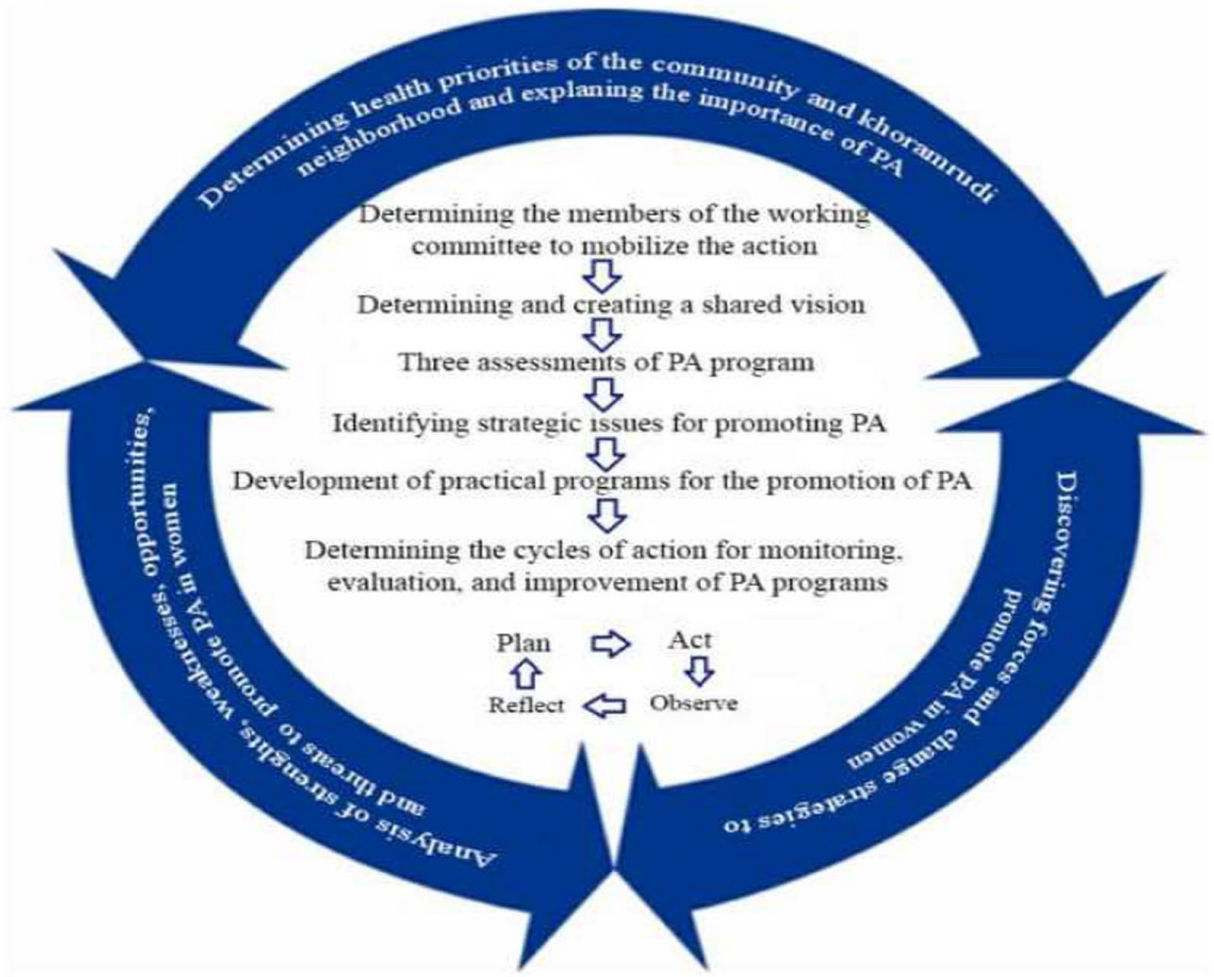
Improving PA among woman living in Khoramroudi neighborhood based on the adjusted MAPP model [30]

Data collection in this phase is described in Table 1. The recommendations of all stakeholders in line with our evidential findings were used to promote PA throughout. Directed content analysis was used to analyze the data [25]. After applying changes to this model based on the objectives of AR and obtaining written consent from the model designers, the model was named; the adjusted MAPP model (Fig. 1).

#### Structural explantation

Data collected and analysed within this phase as detailed in Table 1 was used to confirm the program’s desirability and feasibility.

## Results

Results were captured in relation to the four phases of action research described as (1) exploratory; (2) action; (3) monitoring and evaluation and (4) reflection. Here, we report the qualitative and quantitative results from each of these phases.

### First phase (exploratory)

Based on the results of FGDs, the program's vision was co-created to inspire the women of Tehran to engage in PA for 30 min with a moderate intensity for 5 days or more per week, or 20 min of intense PA for 3 days or more per week based on current recommendations. The co-created name for the program was “BELIEVE”.

Based on the results of the urban HEART project, FDGs and an international review of the literature, insufficient PA was agreed as a priority for intervention within the Khoramroudi neighborhood. The prevalence of insufficient PA in some countries, such as Iraq, Malaysia, Turkey, Lebanon, and Saudi Arabia is similar to prevalence in Iran [31]. According to the urban HEART project, the prevalence of PA in men and women in the city of Tehran has been reported as 24.3% and 20.5%, respectively [3]. In district 2 of Tehran, prevalence has been reported at 23.7% and 25.3% in men and women respectively [3]. The present study of PA in women in the Khoramroudi neighborhood found the mean age and body mass index of women to be 41.7 years and 26.066 kg/m^2^, respectively. Also, the majority of participants had a diploma and identified as married housewives. The PA measured (77.8%) was less than 150 min per week among them.

In relation to the identification of barriers and facilitators of PA in women, the construct of exercise milieu and life enhancement were related to the level of PA more than other constructs. Also, the construct of preventive health and lack of family encouragement had the lowest score when compared to other constructs. In total, 19 participants with an average age of 45.36 years participated in the FGDs and individual interviews. They each had a high school diploma and self-identified as housewives. The barriers and facilitators of PA were placed under three categories; intrapersonal factors (Life enhancement, physical performance, psychological outlook, physical exertion, and lack of PA due to health status and concerns); interpersonal factors (Social interaction (Tendency to do PA in the group), encouragement by family members and friends, necessity to have effective and ethical trainer, life responsibilities and obligations, and cultural and religious beliefs of the society); and the environmental factors (Physical factors and organizational and structural factors) [32].

In relation to identifying the positive and negative forces for change, these included existing intra-organizational barriers and constraints, extra-organizational threats, existing intra-organizational facilities and facilitators and extra-organizational opportunities. Some barriers and constraints related to a lack of awareness about the health centers and the geographical location of sports clubs, the operating time of the sports clubs, the large number of cars in the neighborhood, and a lack of sports facilities in parks in the neighborhood. The existence of the Narejestan (Women only) park and several other women only parks in the neighborhood, cooperation of several local educated women and members of the neighborhood with the research, and the short distance between the health center and sports clubs were referred to as facilitators of PA.

In identifying strategic issues for promoting PA, our systematic review identified strategies including raising awareness about the local sports facilities available, holding FGD sessions or educational courses to teach problem solving skills, overcoming barriers, and goal setting, the need to design bespoke programs, perceived barriers and facilitators of PA, and the need to design programs based on the stages of behavior change in each individual [33].

Our FDGs designed to extract strategies to promote PA based on the MAPP process resulted in strategies classified under 7 categories. These were (1) available information; (2) the condition of the trainer; (3) suitability of facilities and equipment; (4) the raising of individual awareness and skills; (5) the use of a sports coach; (6) the value of group exercise and (7) increased perceived sensitivity to do exercise. Strategies selected as priorities included the use of a sports coach, raising awareness about the existing local sport facilities, and the facilitation of cognitive behavioral therapy classes.

### Second phase (action) and third phase (monitoring and evaluation)

To promote PA in women during the ‘action’ phase of this research, 3 interventions were co-created and implemented over a 3-month period. These were: (1) utilization of sports assistants; (2) Local health promotion and the dissemination of an informational, motivational, and culturally competent booklet entitled "Educational content for sport assistants" (3) Group-based cognitive behavioral therapy (Weekly sessions lasting 90 min).

#### Quantitative evaluation (conducted with cohort 1)

According to the categorization of the IPAQ, people were classified according to MET scores into 3 groups relating to low activity (MET less than 600); moderate activity (MET between 600 and 3000); and high activity (MET more than 3000). Based on this categorization, individuals experienced a change from a low level of PA to a moderate increase during the third month of the intervention period. Comparisons in relation to sitting and vehicle use were not statistically significant before, 1 month, and 3 months after the intervention period using the repeated measures ANOVA test. At the end of the third month after the intervention, only 5 people participated in intensive PA, where other participants engaged in moderate PA.

**Fig. 2:**
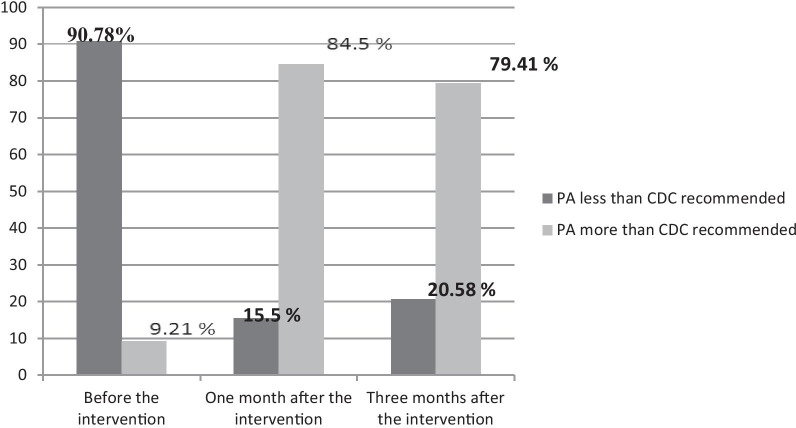
Comparison of the PA before the intervention, and 1 month and 3 months after the intervention among women

The main goal of the BELIEVE program was for participants to engage in PA for 30 min with a moderate intensity for 5 days or more per week, or 20 min of intense PA for 3 days or more per week based on current recommendations [2] (Fig. 2). The amount of PA between the three measurements was compared using the Cochran test, which showed statistical significance (*P* < 0.001). Comparisons between the two measurements were taken using the McNemar test, where the Bonferroni's Correction was set as *P* < 0.0167 in the analysis.

In terms of where participants engaged in PA, 35 people (46.05%) engaged at the local sports club, 18 (23.68%) engaged in individual exercise, 8 (10.8%) engaged in aerobic exercise and 15 (19.73%) engaged in a combination of sports at the sports club and individual exercise. Those individuals who were practicing individual exercise had treadmills at home or walked in the city park. Also, 10 participants purchased digital copies and exercised at home. The quantitative results of this evaluation relate to data collected between August 2014 and February 2015 and are presented in Table 2.

**Table 2:** Frequency distribution of presented strategies and beginning of exercise in the women of Khoramroudi neighborhood

Type of strategy		Start of exercising (Individual or group exercise in club) (Number)
Sport assistants	Lectures in mosques and Quranic interpreting sessions (at least once)	13
Knowledge about the sports facilities	Text message (n = 250)	1
	Posters (n = 1200)	6
	Banner (n = 2)	37
Cognitive behavioral therapy^a^	Two courses	25
Attracting participation by athletes	–	10
Participating women from previous phases of study	–	9
Total		76

^a^Since the group had entered into the action cycle through other interventions, they had already been calculated once in other cases. As such, they were not counted again in this total sum

The mean age and body mass index of the participants was 47.73 years (28–65 years old) and 27.51 kg/m^2^, respectively. They predominantly (34.21%) had high school education and a diploma, self-identified as housewives (82.89%) and were married (97.36%). The repeated measures ANOVA showed a significant difference between the total score of PA before the intervention in terms of energy consumption (MET-minutes/week), and scores taken 1 and 3 months after the intervention (*P* < 0.001). This difference was related to the comparison before the intervention (1251.9 ± 12.13/13) and 1 month later (2667 ± 1759.31) (*P* < 0.001), then before the intervention and 3 months later (2489 ± 1659.41), (*P* < 0.001). Compared to 1 and 3 months later, this difference was not significant (*P* = 0.27). For the comparison of three measurements to reduce the first type error, Bonferroni’s correction was used and *P* < 0.0167 was considered statistically significant.

#### Qualitative evaluation (conducted with cohort 2)

Themes identified from the women’s experiences during the evaluative phase included their exercise achievements (Body flexibility, fitness, recovery as the exercise achievement, improving work quality, effect of exercise in improving mood and feeling of well-being), and their exercise difficulties (Exercise environment, cultural or religious beliefs, lack of social support, health and exercise concerns, desire for comfort, and relaxation and neglect). Themes also identified included facilitators of exercise (Cognitive behavior therapy classes, music, controlling continuity of exercise, effective and ethical coaches, and action plan interventions), and progressive movements for improving the quality and continuity of exercise (Institutionalization of exercise in life, institutionalization of action strategies in health center, doing exercise at home, holding training classes, and increasing awareness).

### Fourth phase (reflection) including process and structural explantation

Our reflective activities resulted in observations that PA in 140 women aged 18–65 years in the Khoramroudi neighborhood improved through engagement with our co-created interventions. Facing barriers to the implementation of strategies also led to new solutions such as using digital copies to facilitate exercise at home. Our reflective phase further led to the development of a process template. Thus the steps and measures taken to explore and implement strategies for promoting PA in women led to the presentation of a modified MAPP process, which differed from the first MAPP model (Fig. 1). Moreover, improvements were made to enhance the exercise environment, including the repairing of the park and the replacing of sports equipment, and the use of local stakeholders led to improved communication in the community. Nevertheless, funding remained a permanent challenge throughout.

## Discussion

Findings showed that mobilizing for action through planning, partnerships, the involvement of local residents, and the use of existing facilities improved PA in women overall. In the present study, the promotion of PA and behavior changes were made possible through the organization of expert forces in the fields of health promotion and PA, interaction with stakeholders inside and outside of the organization, and by attracting the participation of women in the district. This was despite the lack of structural interactions in place between the district and academic institutions/organizations previously.

Similarly, Akbari et al. [34], in justifying the need for structural changes for the effective management of health services in Iran and its barriers, described the importance of providing such opportunities for participation by all stakeholders and the establishment of intergovernmental committees for decentralized management of health sectors. Khorasani et al. [35] likewise described how a lack of such interactions between the professionals and interdisciplinary staff may inhibit AR. The present AR study provides one example of how to overcome this.

This study also showed how paying attention to people's wishes, interests and desires can lead to the development of better interventions and results in research. The continuation of health promotion programs requires not only the identification of problems and the designing of program-based interventions, but also the meeting of challenges in people’s individual lives [36]. Making changes to a person’s level of PA at the macro level is complex, and related to many intrinsic, socio-cultural, environmental, political and financial variables [37]. As such, in designing programs that improve PA, a set of complex and relevant social, medical, economic, familial, physical, and behavioral factors should be considered [38]. Based on the available evidence, the best way to promote PA is by involving societies and individuals [39–41]. According to Whitehead’s study, an individuals’ level of control over their behavior increases their internal motivation. Evidently, when people have the right to choose, they tend to continue that behavior [42].

The simultaneous presence of several strategies may have increased the chances of success in the BELIEVE program. Yet it is likely that, some interventions within the program will have more lasting effects than others, as when multiple interventions are combined, synergic effects may enhance the impact of interventions on PA over time and lead to more stable behaviors [43]. In the program BELIEVE, increasing information in Khoramroudi neighborhood, in addition to acquainting individuals with a local sports club, led to increasing women's awareness about the health center and its services and cognitive behavior therapy courses. In addition, educational approaches were effective in attracting women to increase their PA. This may somewhat explain the change noted in the level of PA as participants ‘understanding of what PA consitutues increases throughout the programme'. Monitoring participation and follow-up by the researcher and other participating women led to greater effectiveness of the interventions.

The findings of the present study showed that approaches based on personal training and individual behavior changes increased PA less than when the changes are made in the environment. Therefore, it will also be necessary to develop strategies for the promotion of PA that involve general public and high-risk groups to sustain social-cultural changes [44]. Due to the fact that the study approach is action research, identifying the problems and needs of individuals and designing a program based on it led to the presentation of different approaches to promote PA. Some people may enjoy individual exercise, and some may improve their PA by receiving social support from a friend or companion. There is strong evidence that individually designed social support and behavior change programs can increase participation in PA [45] (Kahn et al. [39]). In another study, it was also shown that the formation of groups of women and guidance by facilitators to do exercise led to increased PA [46]. The greater the participation of stakeholders in all levels of program design, implementation and evaluation, the better the chances of program success in promoting PA.

The gradual and continuous process of planning and participatory decision-making by all stakeholders was guided by the conceptual framework of AR in this study. The plans and strategies of AR after considering the existing resources, facilities and conditions, and also changes in the structural capacities of the health center according to the women’s needs made it easy to face the challenges and obstacles. Significantly, the strategies for promoting PA in the Khoramroudi district were fueled by the participating women and the selection of strategies. This is encouraging for those looking to co-create similar programs in future, and/or the sustainability and spread of the BELIEVE program elsewhere.

Although AR attempts to promote behaviors in proportion to the resources and facilities of the community and the needs of participants, the existence of barriers at the environmental and organizational levels can lead to greater use of individual and interpersonal interventions. On this point, Cuaderes et al. [47] considered individual motivation and interest to be the most important determinants of the prevalence and severity of PA. This is also reflected in the literature elsewhere [47], as individuals with a high level of motivation encourage themselves without physical support [48]. As such, future studies could usefully evaluate the motivation of their target populations to engage in PA prior to the development of any future interventions.

The individual and collective changes made during this study appear in a linear format. However, their implementations were not linear. The planning process is usually a non-linear process, influenced by critical analysis and feedback [49]. AR, over time and with increased experience is promoted as an evolutionary and progressive process [50]. In the present study, during the implementation period, a linear programming model using the MAPP process in conjunction with the action cycle was used (Planning, implementation, and evaluation cycle). Encouragingly in this case during periods of recurrence, AR gradually and successfully led to the promotion of PA among women in Tehran.

## Strength and limitations

Our mixed methods approach has enabled a detailed evaluation of a unique and replicable project grounded in action research. Yet our results cannot be generalized. However, the introduction of the conceptual framework MAPP and the ‘BELIEVE’ program, which contains detailed explanations of the methods of executing role expansion, including structural considerations of the partnerships involved may increase the transferability of the study’s results to similar fields. Since the current study was conducted in the Khoramroudi neighborhood (One of the above-average areas in terms of socio-economic status in the areas affiliated to Tehran Municipality), the perceived barriers of insufficient PA and the strategies presented may be more generalizable in similar settings.

## Conclusion

Through the use of the MAPP conceptual framework, AR, a situational analysis of the field of research, participation of stakeholders, and an identification of the barriers and facilitators of PA, this study explored strategies to promote PA among women in Tehran. Adherence to the principles of the MAPP process, attracting the participation of women, maximizing the potential usage of the local health center, obtaining executive support from the steering committee and the use of a guiding framework facilitated the planning and implementation of actions overall. In explaining the structure of the program for promoting PA in the Khoramroudi district of Tehran, this article offers a valuable contribution to the literature in this field of research. Future research could usefully explore whether this program may be effective for other women in alternate geographical areas.

## Data Availability

The data that support the findings of this study are available from [Leila Amiri-Farahani] but restrictions apply to the availability of these data, which were used under license for the current study, and so are not publicly available.

## Declarations

### Accordance

All methods were carried out in accordance with relevant guidelines and regulations.
